# Molecular mechanisms and therapeutic strategies in overcoming chemotherapy resistance in cancer

**DOI:** 10.1186/s43556-024-00239-2

**Published:** 2025-01-06

**Authors:** Yixiang Gu, Ruifeng Yang, Yang Zhang, Miaomiao Guo, Kyle Takehiro, Ming Zhan, Linhua Yang, Hui Wang

**Affiliations:** 1 Department of Biliary-Pancreatic Surgery, Renji Hospital, Shanghai Jiao Tong University School of Medicine, Shanghai, 200011 China; 2 Shanghai Key Laboratory of Biliary Tract Disease, Renji Hospital, Shanghai Jiao Tong University School of Medicine, Shanghai, 200011 China; 3 Department of Urology, Fudan University Shanghai Cancer Center, Shanghai, 200032 China; 4 The Core Laboratory in Medical Center of Clinical Research, State Key Laboratory of Medical Genomics, Shanghai Ninth People’s Hospital, Shanghai Jiao Tong University School of Medicine, Shanghai, 200125 China; 5 Arcadia High School, Arcadia, CA 91006 USA; 6 Department of Systems Biology, Beckman Research Institute, City of Hope, Monrovia, CA 91016 USA

**Keywords:** Chemotherapy resistance, Molecular mechanisms, Apoptosis evasion, Cancer stem cells, Epigenetic modifications, Targeted therapy

## Abstract

Cancer remains a leading cause of mortality globally and a major health burden, with chemotherapy often serving as the primary therapeutic option for patients with advanced-stage disease, partially compensating for the limitations of non-curative treatments. However, the emergence of chemotherapy resistance significantly limits its efficacy, posing a major clinical challenge. Moreover, heterogeneity of resistance mechanisms across cancer types complicates the development of universally effective diagnostic and therapeutic approaches. Understanding the molecular mechanisms of chemoresistance and identifying strategies to overcome it are current research focal points. This review provides a comprehensive analysis of the key molecular mechanisms underlying chemotherapy resistance, including drug efflux, enhanced DNA damage repair (DDR), apoptosis evasion, epigenetic modifications, altered intracellular drug metabolism, and the role of cancer stem cells (CSCs). We also examine specific causes of resistance in major cancer types and highlight various molecular targets involved in resistance. Finally, we discuss current strategies aiming at overcoming chemotherapy resistance, such as combination therapies, targeted treatments, and novel drug delivery systems, while proposing future directions for research in this evolving field. By addressing these molecular barriers, this review lays a foundation for the development of more effective cancer therapies aimed at mitigating chemotherapy resistance.

## Introduction

In 2020, an estimated 19.3 million new cancer cases were reported globally, with approximately 10 million deaths attributed to cancer and related complications [1]. Cancer has become a leading cause of mortality worldwide, posing a significant challenge to life expectancy [2]. Radical resection remains the primary treatment option for many malignant tumors. However, due to the aggressive and often asymptomatic nature of some cancers, early diagnosis is difficult, leading to missed opportunities for timely intervention during the most effective treatment windows [3, 4]. This often reduces the potential for curative outcomes.

In this context, chemotherapy has emerged as a pivotal treatment strategy [5]. It is classified based on therapeutic goals into neoadjuvant, adjuvant, curative, and palliative chemotherapy [6–8]. The use of chemotherapy has proven effective in treating not only solid tumors but also hematologic malignancies, enhancing preoperative conditions and lowering postoperative recurrence rates, thus improving overall survival [9]. However, despite these advancements, chemotherapy resistance remains a critical obstacle to achieving effective treatment outcomes. This resistance can be divided into intrinsic resistance, present before treatment begins, and acquired resistance, which develops during the course of therapy [10]. Early identification of these resistance types is essential for optimizing treatment strategies and improving clinical outcomes [11]. Yet, the molecular basis of chemotherapy resistance remains incompletely understood, complicating efforts to overcome it.

**Fig. 1:**
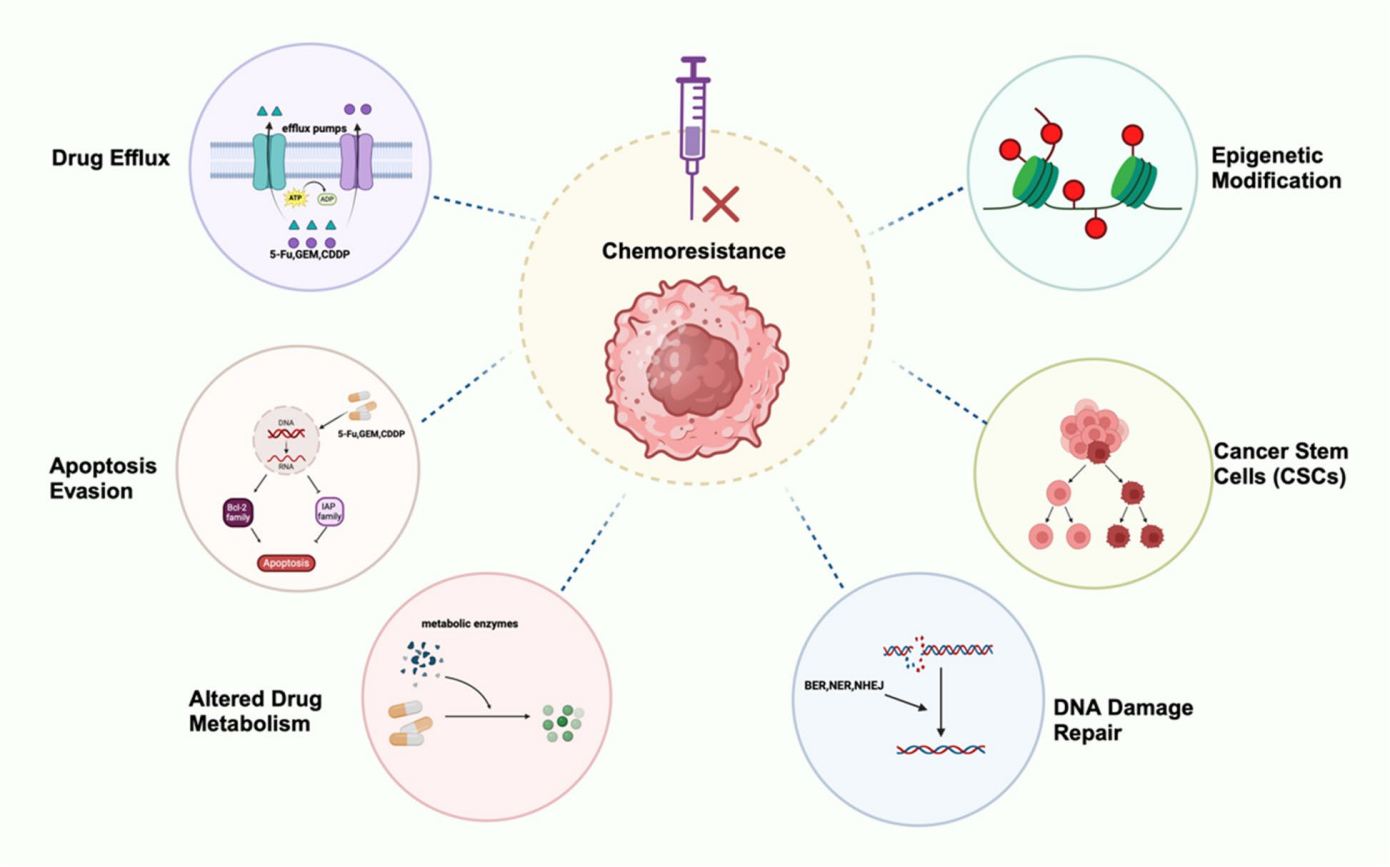
General mechanisms of cancer chemoresistance. Upon exposure to chemotherapeutic agents, tumors initiate multiple mechanisms that mediate chemoresistance. Here, we delineate the molecular pathways involved in cancer chemoresistance, including: drug efflux, DNA damage repair, apoptosis evasion, epigenetic modifications, Altered Drug Metabolism, Cancer Stem Cells (CSCs)

This review aims to explore the complex molecular mechanisms driving chemotherapy resistance, including drug efflux, enhanced DNA damage repair, apoptosis evasion, epigenetic modifications, and the role of cancer stem cells. (Fig. 1) Unlike previous reviews, we not only summarize these mechanisms comprehensively but also highlight recent advances, such as the role of epigenetic modifications in chemoresistance [12, 13]. Additionally, the review outlines resistance mechanisms across major cancer types and identifies key therapeutic targets. Finally, we discuss strategies to overcome resistance, with a focus on emerging therapies. By addressing these molecular barriers, this review provides a foundation for developing more effective approaches to combat chemotherapy resistance in cancer treatment.

## General mechanisms of chemotherapy resistance

Chemotherapy resistance in cancer is driven by a network of fundamental molecular mechanisms, including drug efflux, enhanced DNA damage repair, apoptosis evasion, epigenetic modifications, cancer stem cell dynamics, and alterations in intracellular drug metabolism. These mechanisms are highly interconnected and mutually influential, forming a complex landscape that remains only partially understood. This section systematically describes these molecular pathways, highlighting their roles in the development of chemotherapy resistance.

### The excretion of chemotherapy drugs

The accelerated efflux of chemotherapy drugs from cancer cells significantly reduces their intracellular concentrations, contributing to chemotherapy resistance. This drug efflux is primarily facilitated by ATP-binding cassette (ABC) transporters, including ABCB1, ABCG2, and ABCC1, which are expressed in various organs such as the liver, intestines, kidneys, and brain [14–16]. These transporters use energy from ATP hydrolysis to expel a wide range of substrates, including chemotherapeutic agents, thereby decreasing the cellular exposure to these drugs.

One of the most well-studied efflux transporters is P-glycoprotein (P-gp), encoded by the ABCB1 (MDR1) gene, which is located on the apical membranes of various epithelial cells [17]. P-gp plays a crucial role in removing chemotherapeutic drugs from cancer cells, thus preventing their accumulation. Overexpression of MDR1 has been observed in gallbladder cancer (GBC) and is associated with increased resistance to drugs such as gemcitabine and 5-fluorouracil (5-FU) [18–21]. Elevated MDR1 expression has also been reported in other cancers, including colorectal cancer, breast cancer, osteosarcoma, and pancreatic cancer, where it contributes to multidrug resistance [22]. Another significant efflux pump is the Multidrug Resistance-associated Protein 1 (MRP1), part of the ABCC family. MRP1 plays a pivotal role in multidrug resistance by exporting chemotherapeutic agents out of cells [23, 24]. Targeting MRP1, for instance through the miR-145-MRP1 axis, shows potential in reversing chemoresistance [25]. In GBC, intracellular glutathione (GSH) can bind to cisplatin, forming GS-platinum complexes that are expelled by MRP1. Strategies that downregulate MRP1 or deplete GSH enhance the cytotoxicity of cisplatin, suggesting potential therapeutic approaches [26].

Breast Cancer Resistance Protein (BCRP), encoded by the ABCG2 gene, is another critical efflux transporter involved in drug resistance. BCRP protects cells by exporting toxic substances, including chemotherapy drugs, and is expressed in tissues like the intestines, bile ducts, placenta, blood-testis barrier, and blood–brain barrier [27]. Studies have linked increased BCRP expression to enhanced chemotherapy resistance [28, 29]. Notably, the overexpression of BCRP has been observed in metastatic cancer cells, leading to intrinsic resistance, even without prior exposure to chemotherapy [30].

In addition to ABC transporters, copper-transporting ATPases such as ATP7A and ATP7B help maintain intracellular copper balance but can also sequester platinum-based drugs like cisplatin. This sequestration reduces the drugs' efficacy and contributes to chemotherapy resistance [31]. Exploring the roles of ATP7A and ATP7B in GBC may provide insights into overcoming platinum-based drug resistance [32]. Although significant progress has been made in understanding drug excretion mechanisms, further research is required to fully elucidate the pathways involved in chemotherapy resistance, particularly in cancers like GBC. Developing therapeutic strategies targeting these efflux mechanisms may help overcome resistance and improve treatment outcomes.

### DNA damage repair

When exposed to chemotherapeutic agents such as topoisomerase inhibitors, alkylating agents, or DNA cross-linking drugs like cisplatin and cyclophosphamide, tumor cells undergo various forms of DNA damage. In response, cells activate a network of DNA damage response (DDR) pathways to maintain genomic stability and integrity [33]. The most commonly observed DNA repair mechanisms include direct reversal of DNA lesions, base excision repair (BER), nucleotide excision repair (NER), and mismatch repair (MMR). For repairing DNA double-strand breaks (DSBs), two key pathways are involved: homologous recombination (HR) and non-homologous end joining (NHEJ) [34].

The efficiency of DNA damage repair plays a critical role in both tumor development and the sensitivity of cancer cells to chemotherapy. For example, cells with activated RAS and PI3K signaling pathways produce elevated levels of reactive oxygen species (ROS), which cause oxidative DNA damage. These cancer cells activate the BER pathway to repair oxidative lesions. However, this increased DNA repair capability can paradoxically enhance resistance to chemotherapy drugs like temozolomide and cisplatin [35]. Additionally, studies have shown that after DNA damage, the levels of Dicer, an enzyme involved in RNA processing, are significantly elevated. This promotes DNA repair through the NHEJ pathway, contributing to chemotherapy resistance in colorectal cancer [36]. This highlights the critical role of DDR pathways in developing chemotherapy resistance across various cancer types.

Overall, enhanced DNA repair mechanisms protect tumor cells from the cytotoxic effects of chemotherapy by rapidly repairing the DNA damage that these treatments induce. Understanding how these pathways function provides insight into potential targets for overcoming resistance. Inhibitors targeting specific components of DNA repair pathways, such as PARP inhibitors, have shown promise in sensitizing resistant cancer cells to chemotherapy by disrupting their ability to repair DNA damage effectively.

### Apoptosis evasion

**Fig. 2:**
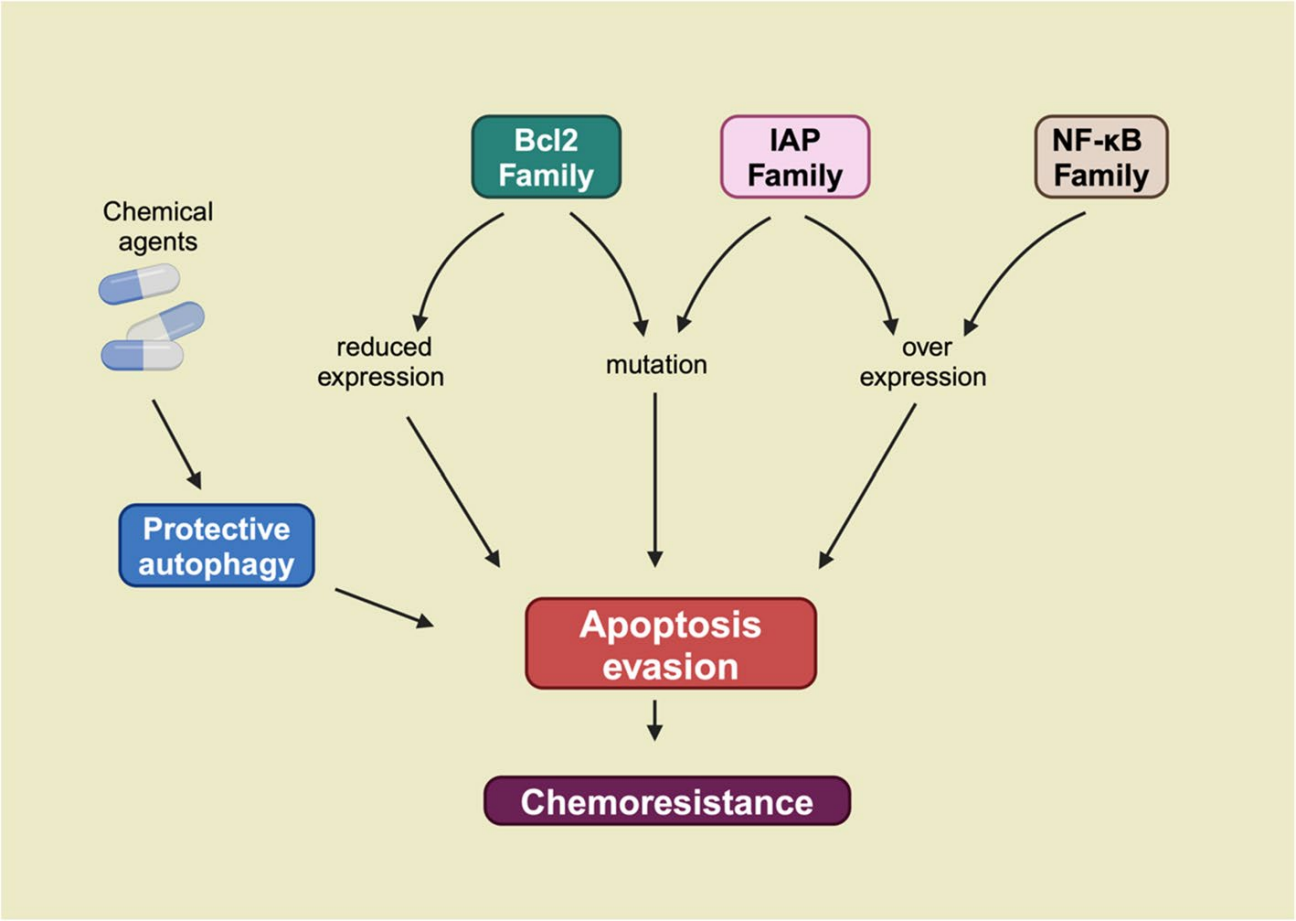
Apoptosis Evasion as a Mechanism of Chemoresistance. Chemotherapeutic intervention in tumor cells activates the intrinsic protective autophagy system. Concurrently, dysregulated expression and mutations in Bcl-2, IAP, and NF-κB family proteins impair chemotherapy-induced apoptosis in tumor cells. These factors collectively enable apoptosis evasion, leading to enhanced chemoresistance

Apoptosis, or programmed cell death, is a tightly regulated process essential for eliminating damaged or harmful cells. Chemotherapeutic agents often induce apoptosis in cancer cells to exert their therapeutic effects [37, 38]. However, cancer cells frequently develop mechanisms to evade apoptosis, contributing significantly to chemotherapy resistance [39]. This evasion is driven by disruptions in apoptotic pathways, protective autophagy, and the overexpression of anti-apoptotic proteins. (Fig. 2).

One key mechanism by which cancer cells avoid apoptosis is through protective autophagy. Autophagy, a cellular self-degradation process, is activated in response to stress and serves as a survival mechanism in tumor cells [40]. Chemotherapy often triggers autophagy in cancer cells, which then inhibits apoptosis, reducing the effectiveness of treatment [41]. For instance, in cisplatin-resistant ovarian cancer cells, autophagy alleviates endoplasmic reticulum (ER) stress, thereby preventing mitochondrial-dependent apoptosis [42]. Similarly, in gallbladder cancer (GBC), nutrient deprivation, such as glucose starvation, triggers autophagy via the IL-6/STAT3 pathway, reducing apoptosis and increasing resistance to gemcitabine [43]. Similarly, in pancreatic cancer, Girdin activates protective autophagy by interacting with the autophagy-related protein p62/SQSTM1, which inhibits apoptotic pathways and promotes chemoresistance [44]. Evidence suggests that inhibiting autophagy may enhance the effectiveness of chemotherapy [45, 46]. Blocking autophagy with inhibitors like chloroquine has shown promise in restoring chemosensitivity.

In addition to autophagy, the dysregulation of apoptosis-related pathways plays a major role in chemotherapy resistance. The B-cell lymphoma 2 (Bcl-2) family of proteins regulates the mitochondrial outer membrane permeabilization, a key step in apoptosis. Overexpression of anti-apoptotic members of the Bcl-2 family, such as Myeloid Cell Leukemia-1 (Mcl-1) and Bcl-2, has been linked to chemotherapy resistance in several cancers, including leukemia, lung cancer, and breast cancer [47, 48]. In ovarian cancer, for example, cell lines with high Bcl-2 expression show increased resistance to chemotherapy [49]. Conversely, the knockdown of Mcl-1 restores chemosensitivity [50].

Mutations or reduced expression of pro-apoptotic proteins also contribute to resistance. For instance, mutations in the Bcl-2-associated X protein (BAX) gene, a pro-apoptotic protein, have been associated with colorectal cancer and therapeutic resistance [51]. Similarly, the downregulation of BAX and Phorbol-12-myristate-13-acetate-induced protein 1 (NOXA), other pro-apoptotic proteins, has been linked to resistance in acute myeloid leukemia (AML) [52–54]. Another important group of proteins involved in apoptosis regulation is the inhibitor of apoptosis proteins (IAPs). IAPs inhibit apoptosis by blocking caspase activity, thereby promoting cancer cell survival. Overexpression of IAP family members, such as X-linked inhibitor of apoptosis protein (XIAP), Survivin, and cIAP, has been associated with resistance to cisplatin and other chemotherapeutic agents [55]. Strategies targeting IAPs, such as second mitochondria-derived activator of caspases (SMAC) mimetics, are currently being explored to overcome apoptosis evasion [56]. Additionally, aberrant activation of the nuclear factor kappa-B (NF-κB) signaling pathway promotes apoptosis resistance in several cancers. NF-κB dysregulation has been shown to contribute to resistance to drugs like doxorubicin, cisplatin, and paclitaxel [57]. In GBC, co-administration of NF-κB inhibitors with chemotherapy has been found to enhance the sensitivity of cancer cells to gemcitabine [58].

In conclusion, evasion of apoptosis is a complex and multifaceted process in cancer cells that significantly contributes to chemotherapy resistance. Targeting key proteins involved in autophagy, anti-apoptotic signaling, and apoptosis regulation offers promising strategies to enhance the effectiveness of chemotherapy and overcome resistance.

### Epigenetic modifications

**Fig. 3:**
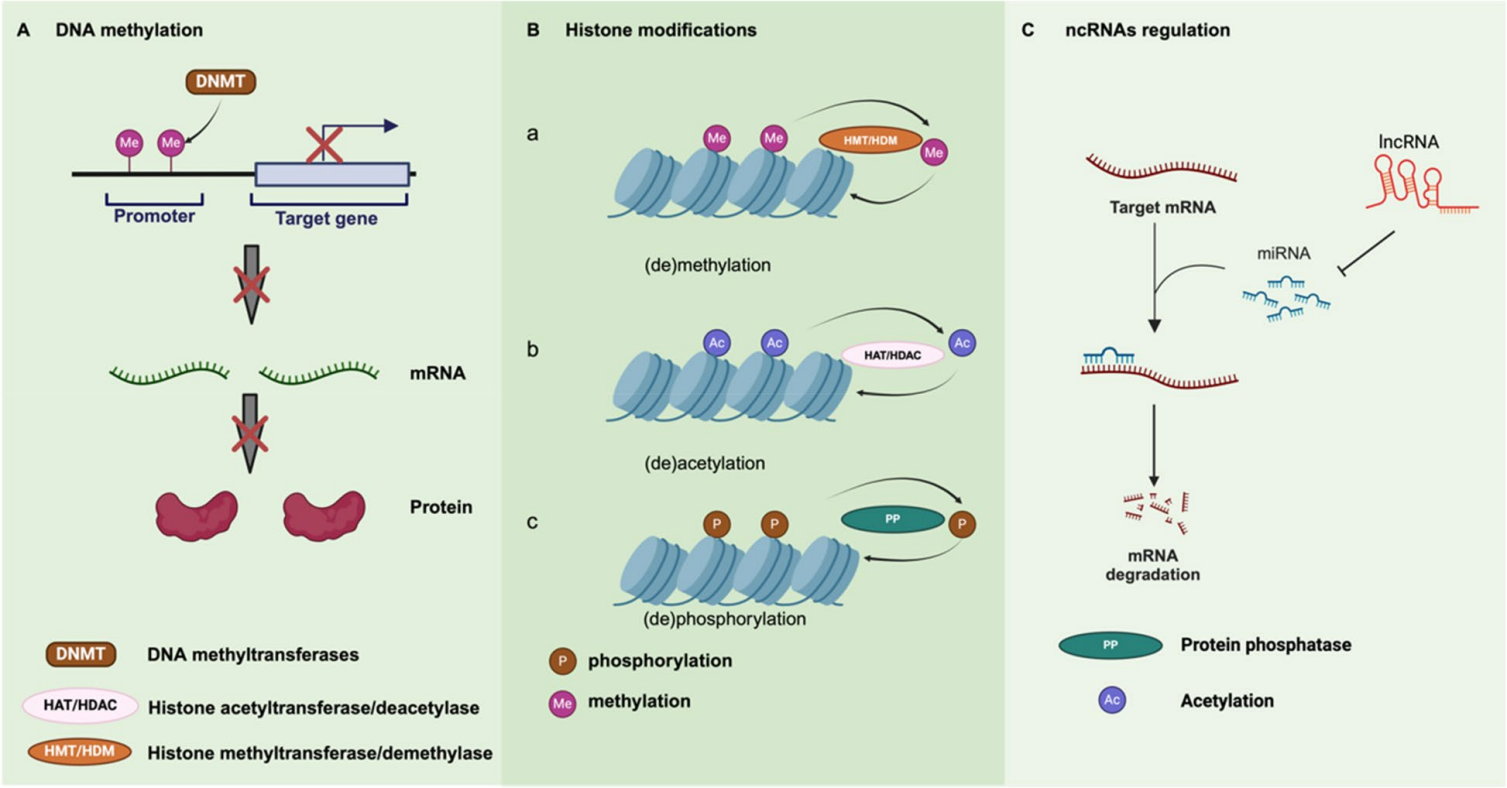
Overview of epigenetic modifications involved in cancer. (1) DNA methylation. Through the catalytic activity of DNMTs, cytosine residues are selectively methylated, producing 5-methylcytosine (5-mC). Methylation in promoter regions effectively downregulates the transcription of target genes, ultimately restricting the translation of mRNA into proteins. (2) Histone modifications. (a) (de)methylation. Histone methylation and demethylation, mediated by HMTs and HDMs, indirectly modulate chromatin accessibility and gene expression by altering the binding affinities of other epigenetic regulators. (b)(de)acetylation. Histone acetylation and deacetylation, catalyzed by HATs and HDACs, induce structural alterations in chromatin, thereby modulating gene expression. (c)(de)phosphorylation. Through the catalytic activities of PKs and PPs, histones undergo phosphorylation and dephosphorylation, facilitating rapid adjustments in chromatin structure and gene expression in response to cellular conditions and demands. (3) ncRNAs regulation. miRNA binds to the 3’ untranslated region (3’-UTR) of target mRNAs, typically leading to mRNA degradation or translational repression, which in turn reduces target protein expression. Conversely, lncRNA can act as a “miRNA sponge” by binding to miRNAs, preventing them from associating with their target mRNAs and thus promoting the expression of specific mRNAs

Epigenetic modifications, which are heritable changes in gene expression without alterations to the DNA sequence, play a pivotal role in cancer development and progression. These changes are reversible and primarily occur through mechanisms like DNA methylation, histone modifications, and non-coding RNA regulation. (Fig. 3) Epigenetic alterations are closely linked to chemotherapy resistance by influencing the transcription and expression of key genes involved in drug response [59].

DNA methylation, the addition of methyl groups to CpG dinucleotides, is one of the earliest and most studied epigenetic mechanisms [60]. DNA methylation patterns in tumors often include global hypomethylation and site-specific hypermethylation of tumor suppressor genes [61]. For example, hypermethylation of the Breast Cancer 1 (BRCA1) gene in ovarian cancer cells is associated with increased sensitivity to platinum-based chemotherapy [62]. Conversely, the hypermethylation of genes like TGF-βRI in gastric cancer has been linked to resistance to transforming growth factor-β (TGF-β) signaling [63, 64]. Reversing these methylation patterns may help overcome chemotherapy resistance [65]. Histone modifications, including acetylation, methylation, ubiquitination, and phosphorylation, also regulate gene expression by altering chromatin structure. Histone acetylation, mediated by histone acetyltransferases (HATs) and reversed by histone deacetylases (HDACs), is particularly important in chemotherapy resistance [66]. Acetylation generally leads to chromatin relaxation, enhancing transcriptional activity, while deacetylation condenses chromatin, silencing genes [67, 68]. For example, the overexpression of p300, a HAT, has been associated with gemcitabine resistance in pancreatic cancer [69]. On the other hand, upregulation of HDACs is linked to resistance in various cancers, including melanoma and ovarian cancer [70, 71]. Inhibiting HDACs with histone deacetylase inhibitors (HDACi), such as belinostat, has shown promise in restoring chemotherapy sensitivity [72]. Histone methylation also plays a key role in drug resistance. The enzyme Enhancer of Zeste Homolog 2(EZH2), a methyltransferase, is overexpressed in cisplatin-resistant ovarian cancer cells. Reducing H3K27 trimethylation (mediated by EZH2) enhances sensitivity to cisplatin [73]. Similar findings have been reported in lung cancer, where histone methyltransferases contribute to chemotherapy resistance by regulating cancer stem cell genes, promoting epithelial-mesenchymal transition (EMT), and influencing cell migration [74].

Non-coding RNAs, particularly microRNAs (miRNAs) and long non-coding RNAs (lncRNAs), are also critical regulators of gene expression and have been increasingly implicated in chemoresistance [75]. MiRNAs, small non-coding RNAs that regulate gene expression post-transcriptionally, can act as either tumor suppressors or oncogenes [76]. In gallbladder cancer, miRNAs have been shown to regulate the expression of drug resistance genes [77, 78]. For instance, overexpression of certain miRNAs can suppress genes involved in drug metabolism or apoptosis, contributing to chemoresistance. Long non-coding RNAs (lncRNAs), which are longer RNA molecules that do not encode proteins, also play a role in drug resistance through interactions with various signaling pathways [79, 80]. For example, GBCDRlnc1 has been identified in doxorubicin-resistant gallbladder cancer cells, where it enhances autophagy and promotes drug resistance [81]. Similarly, in cholangiocarcinoma, the lncRNA HOTTIP modulates sensitivity to chemotherapy through the HOTTIP/miR-637/LASP1 axis [82]. (Table 1).

**Table 1:** Chemoresistance mediated by noncoding RNA in GBC

ncRNA type	Expression	Drug	Target	Reference
MicroRNA				
miR-145	down	CDDP	MRP 1	[25]
miR-125 b-5 p	down	CDDP	Bcl2	[83]
miR-31	down	DDP	Src	[84]
miR-205-5p	down	GEM	PRKCE	[85]
miR-218-5p	down	GEM	PRKCE/MDR1	[19]
miR-193a-3p	down	trametinib	KRAS/ERK	[86]
miR-433	down	GEM	miR-433/cyclin M	[87]
miR-223	down	Docetaxel	STMN1	[88]
LncRNA				
GBCDRlnc1	up	DOX	PGK1	[81]
MYLK-AS 1	up	GEM	MYLK-AS 1/miR-217/EZH 2	[89]
HOTTIP	up	GEM/CDDP	HOTTIP/miR 637/LASP 1	[82]

Epigenetic mechanisms offer potential therapeutic targets, as their reversible nature provides opportunities for intervention. By targeting specific epigenetic alterations, such as using DNA demethylating agents or histone deacetylase inhibitors, chemotherapy resistance may be mitigated.

### Altered drug metabolism

Chemotherapeutic agents undergo extensive metabolism within tumor cells, often affecting their therapeutic efficacy. The enzymes responsible for drug metabolism are critical factors in the development of chemotherapy resistance. This process is generally divided into two phases: Phase I metabolism, which involves oxidation, reduction, or hydrolysis by enzymes such as cytochrome P450 (CYP), and Phase II metabolism, where drugs are conjugated by enzymes such as glutathione S-transferases (GSTs) [90]. Cytochrome P450 (CYP) enzymes play a key role in drug biotransformation. Alterations in CYP enzyme levels can significantly affect drug metabolism, leading to resistance. For example, overexpression of CYP1B1, CYP2E1, and CYP3A4 has been observed in non-rhabdomyosarcoma soft tissue sarcomas, contributing to reduced sensitivity to chemotherapy [91–93]. Conversely, decreased CYP expression has been linked to increased chemoresistance in human medulloblastoma cells [91].

The glutathione (GSH) metabolic system also plays a significant role in drug resistance. Elevated levels of intracellular GSH allow cancer cells to neutralize chemotherapy drugs by conjugating them with GSH, facilitating their removal from the cell [94]. This process is particularly relevant in cisplatin resistance, where GSH binds to the drug, inactivating it before it can exert its cytotoxic effects [95]. Overexpression of gamma-glutamyl transferase (GGT), which is involved in GSH metabolism, further enhances resistance by protecting cancer cells from oxidative stress and chemotherapeutic damage [96]. Other metabolic enzymes are also implicated in chemotherapy resistance. Deoxycytidine kinase (dCK), a critical enzyme in the activation of gemcitabine, is often downregulated in gemcitabine-resistant cancer cells. Mutations or reduced expression of dCK have been identified in resistant gallbladder cancer cell lines, where restoring dCK levels can re-sensitize cells to the drug [97–99].

The complexity of drug metabolism, including alterations in both Phase I and Phase II pathways, underscores its significant role in chemotherapy resistance. Targeting key metabolic enzymes such as CYPs, GSTs, or dCK offers potential strategies for overcoming drug resistance and improving the efficacy of chemotherapeutic agents.

### Cancer stem cells (CSCs)

**Fig. 4:**
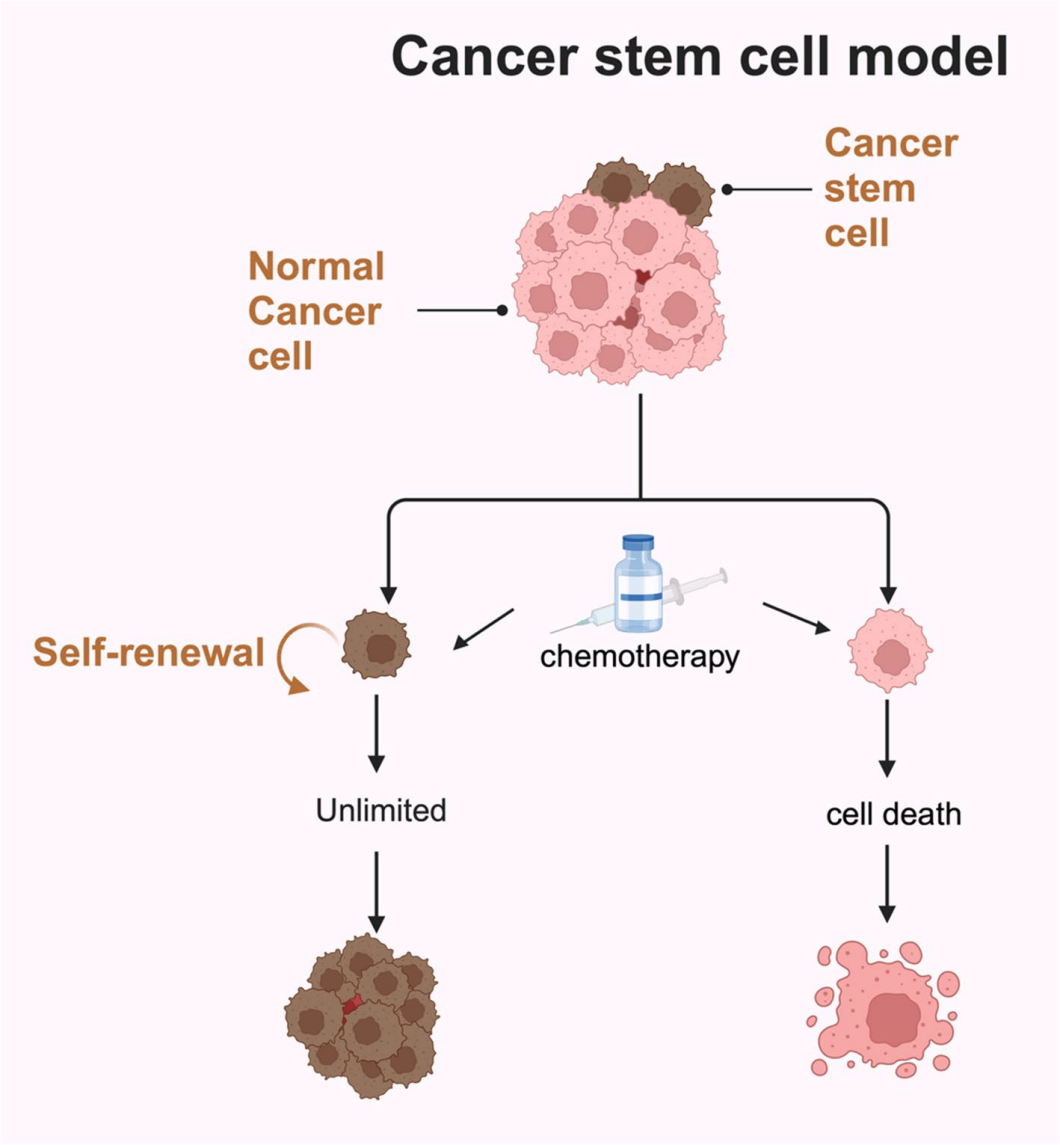
Cancer stem cell model. Tumor tissues contain CSCs, a subpopulation with self-renewing properties. When chemotherapy is applied, typical cancer cells undergo apoptosis, yet CSCs evade these effects, leading to their survival and the possibility of treatment resistance

Cancer stem cells (CSCs) are a subpopulation of tumor cells characterized by their ability to self-renew, differentiate, and initiate tumor formation. These cells are also highly resistant to conventional chemotherapy, which primarily targets rapidly dividing cancer cells. As a result, CSCs often survive treatment, contributing to tumor recurrence and metastasis [100, 101]. (Fig. 4) CSCs express specific surface markers, such as CD133, CD44, and aldehyde dehydrogenase (ALDH), which help identify and isolate them [102].

One of the key mechanisms by which CSCs resist chemotherapy is through drug efflux, facilitated by ATP-binding cassette (ABC) transporters like ABCG2. These transporters actively pump chemotherapy drugs out of CSCs, reducing their intracellular concentration and thereby limiting their cytotoxicity [102, 103]. For instance, CD133 + CSCs in gallbladder cancer exhibit high levels of ABCG2 expression, making them resistant to drugs like gemcitabine and 5-fluorouracil (5-FU) [104]. Similarly, in lung cancer, CD133 + cells show resistance to DNA-damaging agents due to enhanced DNA repair capacity, mediated by increased expression of DNA repair proteins [105]. Enhanced DNA repair mechanisms also contribute to CSCs' ability to survive chemotherapy. CSCs often exhibit upregulated DNA repair pathways, allowing them to quickly resolve chemotherapy-induced DNA damage. For example, XRCC1, a DNA repair protein, is highly expressed in CD133 + GBC cells, contributing to 5-FU resistance [106]. In addition, CSCs are adept at maintaining low levels of reactive oxygen species (ROS), which allows them to evade the oxidative damage caused by certain chemotherapeutic agents. CSCs achieve this by upregulating antioxidant enzymes, which neutralize ROS and protect the cells from apoptosis [107]. This enhanced oxidative stress response helps CSCs survive therapies that induce ROS-mediated cell death [107–110].

Cellular quiescence, a state of dormancy in which cells exit the cell cycle (G0 phase), is another mechanism that protects CSCs from chemotherapy. Most chemotherapeutic agents target rapidly dividing cells, leaving quiescent CSCs unaffected. Once therapy ends, these quiescent CSCs can re-enter the cell cycle, leading to tumor regrowth [111, 112]. The ALDH enzyme, which detoxifies reactive aldehydes and reduces oxidative stress, is also highly expressed in quiescent CSCs, further contributing to their survival and resistance [113, 114]. The ability of CSCs to evade chemotherapy through these mechanisms makes them a critical focus of cancer research. Targeting CSC-specific pathways, such as drug efflux pumps, DNA repair systems, and ROS metabolism, could provide new therapeutic strategies to eliminate CSCs and reduce the likelihood of tumor relapse [115].

In this section, the molecular mechanisms underlying chemotherapy resistance in cancer are comprehensively described. These intricately connected pathways play a pivotal role in the failure of chemotherapeutic interventions, posing substantial challenges in clinical oncology. By gaining a deeper understanding of how these mechanisms contribute to resistance, researchers can more effectively identify novel therapeutic targets, paving the way for advanced treatment strategies and improved outcomes for cancer patients.

## Chemotherapy resistance in major cancer types

While the previous section summarized the primary mechanisms of chemotherapy resistance, the molecular basis of resistance demonstrates considerable heterogeneity across different cancer types. Accordingly, this section systematically examines the resistance mechanisms in several clinically significant malignancies, including lung, breast, colorectal, and prostate cancers, as well as leukemia. These cancers collectively represent a substantial global health burden and are characterized by persistently high rates of therapeutic resistance. By elucidating both the convergent pathways and cancer-specific molecular targets implicated in resistance, this section underscores the complexity of these mechanisms. Critically, a comprehensive understanding of these processes is essential for refining therapeutic strategies and establishing a robust foundation for the development of innovative interventions to overcome resistance.

### Lung cancer

Lung cancer is one of the most common malignancies worldwide, with over 2.2 million new cases and nearly 1.8 million deaths reported in 2020 [116]. It is classified into two main types: non-small cell lung cancer (NSCLC) and small cell lung cancer (SCLC). While curative surgery is often the first-line treatment for early-stage tumors, many patients present with unresectable or advanced disease, requiring systemic therapies such as chemotherapy, targeted therapy, or immunotherapy [117]. In NSCLC, targeted therapies are preferred for patients with specific genetic mutations [118]. However, for those without such mutations, platinum-based chemotherapy remains the standard treatment [119].

Platinum drugs, such as cisplatin, work by forming DNA adducts that interfere with DNA synthesis and transcription, leading to cell death [120]. However, the development of resistance significantly reduces the efficacy of these therapies. Resistance mechanisms include reduced drug accumulation, enhanced DNA repair, and the activation of anti-apoptotic pathways. Reduced intracellular accumulation of platinum is often caused by increased drug efflux or impaired uptake via transport channels, such as copper transporters (CTR1/2) [121, 122]. Additionally, upregulation of efflux pumps like ATP7A/B and MRPs lowers intracellular platinum concentrations, contributing to resistance [123–125].

Enhanced DNA repair also plays a critical role in platinum resistance. Tumor cells with high DNA repair capacity can effectively remove platinum-induced DNA damage. For instance, NPAS2, which stabilizes H2AX (a key enzyme in homologous recombination repair), increases DNA repair activity, decreasing the sensitivity of lung adenocarcinoma cells to platinum drugs [126]. Likewise, the XRCC1/MACC1 complex activates the Akt signaling pathway, contributing to resistance through enhanced base excision repair [127]. In addition, the activation of anti-apoptotic pathways, such as NF-κB and Bcl-2 family proteins like MCL-1, also supports platinum resistance by inhibiting apoptosis [128–130].

Resistance to targeted therapies, particularly epidermal growth factor receptor (EGFR) tyrosine kinase inhibitors (TKIs), is another major challenge in NSCLC. While EGFR-TKIs improve outcomes in patients with EGFR mutations [131, 132], resistance, particularly due to EGFR-T790M mutations, limits their long-term efficacy [133]. Although third-generation EGFR inhibitors like osimertinib have been developed to address T790M-mediated resistance, new mutations, such as EGFR-C797S, have emerged [134–137]. These findings highlight the complexity of resistance mechanisms in lung cancer, underscoring the need for ongoing research to develop more effective treatments.

### Breast cancer

Breast cancer is the most common malignancy worldwide and remains a leading cause of death among women, posing a significant public health concern [138]. It is traditionally classified into molecular subtypes based on the expression of estrogen receptor (ER), progesterone receptor (PR), and human epidermal growth factor receptor 2 (HER2). The main subtypes include hormone receptor-positive (HR +), HER2-positive (HER2 +), and triple-negative breast cancer (TNBC). Current therapeutic strategies are guided by these molecular subtypes [139, 140], although drug resistance mechanisms vary across these categories.

HR + tumors, defined by high expression of ER or PR, represent 70–80% of invasive breast cancer cases [141]. ER activation occurs via two pathways: the classical pathway, where estradiol binds to ER to stimulate proliferation, and the non-classical pathway, where ER is activated by mitogen-activated protein kinase (MAPK) or phosphatidylinositol-3-kinase (PI3K) signaling [142, 143]. This activation promotes gene transcription, driving breast cancer progression. As a result, endocrine therapies targeting the estrogen axis—such as the ER antagonist tamoxifen (TAM) and aromatase inhibitors (AIs) like letrozole and anastrozole—are standard treatments [144]. TAM is commonly used in premenopausal women, while AIs are prescribed for postmenopausal women. However, resistance to endocrine therapy remains a major challenge, with 50–60% of patients experiencing primary or acquired resistance [145]. This resistance is often linked to mutations in the ER ligand-binding domain (LBD) or alterations in signaling pathways. Notably, ESR1 gene mutations, which encode ERα, are rare in primary breast cancer but occur in about 20% of patients with metastatic breast cancer (MBC) who have undergone endocrine therapy [146–148]. The most frequent mutations, Y537 and D538, induce structural changes that reduce sensitivity to TAM and AIs, contributing to resistance [149]. These mutations also enhance cancer cell migration, invasion, and metastasis. Other genetic alterations in ESR1 and aberrations in the PI3K and MAPK pathways further complicate resistance mechanisms [150, 151]. For instance, mutations in PIK3CA or alterations in AKT1 or PTEN, which lead to abnormal activation of the PI3K pathway, are associated with endocrine resistance in ER + breast cancer [152]. Mutations in the MAPK pathway components, such as NF1, KRAS, and BRAF, also contribute to resistance, especially in metastatic cases [153, 154].

Receptor tyrosine kinases (RTKs) regulate pathways like PI3K/AKT and JAK/STAT, and upregulation of fibroblast growth factor receptors (FGFRs) can induce endocrine resistance. HER2 mutations acquired during endocrine therapy in HR + /HER2- patients may promote cross-talk with ER/PR-related pathways, furthering resistance [155]. The advent of cyclin-dependent kinase 4/6 (CDK4/6) inhibitors has improved treatment options, particularly for overcoming resistance to endocrine therapies. CDK4/6 inhibitors block cell cycle progression by preventing the phosphorylation of retinoblastoma protein (RB1), halting tumor cell growth [156, 157]. However, resistance to CDK4/6 inhibitors can occur due to RB1 loss, which allows cells to bypass the CDK4/6 blockade and continue proliferating through alternative pathways, such as E2F [158]. Thus, combining endocrine therapy with CDK4/6 inhibitors is a promising approach, but further clinical trials are necessary to optimize this strategy.

HER2 + breast cancer accounts for 25–30% of all cases and is associated with higher malignancy, recurrence, and poor prognosis. Trastuzumab (Herceptin), a monoclonal antibody targeting HER2, has dramatically improved outcomes in this subtype. However, resistance remains a major challenge [159, 160]. Up to one-third of patients exhibit intrinsic resistance to trastuzumab, and within the first year, 70% may develop acquired resistance [161]. Resistance mechanisms include HER2 gene mutations—such as the HER2-L755S mutation—which promote activation of the PI3K/AKT and MAPK pathways, negating trastuzumab’s efficacy [162, 163]. The presence of p95HER2, a splice variant of HER2, also limits anti-HER2 drug efficacy [164]. Additionally, Mucoprotein 4 (Muc4) can obscure trastuzumab’s binding site on HER2, exacerbating resistance [165]. Aberrant activation of the PI3K/AKT pathway, often through PIK3CA mutations, plays a central role in trastuzumab resistance [166].

Triple-Negative Breast Cancer (TNBC) represents 10–20% of breast cancer cases and lacks expression of ER, PR, and HER2, making it challenging to treat [167, 168]. Current therapies include chemotherapy, radiotherapy, immunotherapy, and targeted therapy, with chemotherapy being the cornerstone of treatment [169]. Despite chemotherapy, resistance remains common, prompting the need for novel therapeutic approaches. BRCA1 and BRCA2 mutations, which impair DNA repair, are significant in TNBC, as tumors with these mutations show heightened sensitivity to poly ADP-ribose polymerase (PARP) inhibitors [170, 171]. PARP inhibitors, such as olaparib, have proven effective in treating BRCA-mutated TNBC [172]. Moreover, mutations in the PI3K/AKT/mTOR pathway are the second most common in TNBC, occurring in 25–35% of cases [173]. Targeting this pathway, along with the androgen receptor (AR) pathway—which is expressed in 10–50% of TNBC cases—offers potential therapeutic avenues [174, 175]. The AR signaling pathway also exhibits significant cross-talk with the PI3K/Akt and MAPK pathways, creating a complex regulatory network that influences tumor biology. Additionally, AR interacts with the Wnt/β-catenin pathway, which plays a critical role in the initiation and progression of TNBC [176, 177]. Furthermore, the AR pathway promotes the transcription of genes involved in epithelial-mesenchymal transition (EMT), increasing the invasiveness and metastatic potential of cancer cells. Therefore, targeting the AR signaling pathway represents a promising therapeutic strategy for TNBC [178].

Multiple resistance mechanisms exist across breast cancer subtypes, driven by complex interactions between genetic mutations, signaling pathways, and tumor microenvironment factors. Despite advances in targeted therapies, the development of drug resistance continues to limit treatment efficacy. Ongoing research into these resistance mechanisms and large-scale clinical trials are essential for the development of precision medicine approaches, which will help to overcome therapeutic resistance and improve patient outcomes.

### Colorectal cancer

Colorectal cancer (CRC) is the third most prevalent cancer globally and the second leading cause of cancer-related mortality, with 1,142,286 new cases reported in 2022 [138]. While surgery remains the primary treatment for CRC, the identification of key molecular targets involved in CRC pathogenesis and progression has led to the development of targeted therapies, offering promising prospects for improving patient outcomes [179]. Notable advancements include therapies targeting Kirsten rat sarcoma viral oncogene homolog (KRAS) mutations, such as KRAS inhibitors, and EGFR inhibitors like cetuximab [180, 181]. However, drug resistance poses a significant challenge to the effectiveness of these therapies, particularly resistance associated with KRAS and EGFR inhibitors.

KRAS mutations are among the most common oncogenic mutations in human cancers, present in approximately 35% to 49% of CRC cases, with the G12C mutation accounting for about 11% of all KRAS mutations. Recently, covalent inhibitors targeting the G12C subtype have shown promise in preclinical and clinical studies, becoming a focal point of KRAS inhibitor research [182]. However, as with other therapies, resistance remains a critical issue in clinical applications. Acquired resistance often arises from secondary mutations in the KRAS gene following treatment with KRAS G12C inhibitors. Genomic analyses of pre- and post-treatment samples from patients—including those with non-small cell lung cancer, CRC, and appendiceal cancer—revealed secondary KRAS mutations (such as G12D/R/V/W, G13D, Q61H, R68S, H95D/Q/R, or Y96C) and KRAS amplification as contributors to resistance. The activation of KRAS requires upstream signaling factors to engage downstream pathways, and reactivation or alteration of these factors can also drive resistance [183]. Notably, reactivation of receptor tyrosine kinase (RTK) signaling, particularly EGFR, plays a crucial role in resistance to KRAS G12C inhibitors in CRC. This mechanism may be due to the inherent RTK dependency in CRC and the reactivation of downstream effectors [184]. Combination therapies targeting both EGFR and KRAS G12C have shown promise in overcoming this resistance [185].

The MAPK pathway, a downstream component of the RAS signaling cascade, also plays a pivotal role in resistance to KRAS-targeted therapies. Feedback activation of this pathway can result in resistance to inhibitors targeting specific KRAS alleles [186, 187]. Additionally, epithelial-to-mesenchymal transition (EMT) may contribute to resistance against KRAS G12C inhibitors in CRC. EMT can alter signaling pathways upstream and downstream of RAS, reducing the effectiveness of KRAS inhibitors on the RAS signaling network [188].

In approximately 60% to 80% of CRC cases, EGFR is overexpressed, making it a critical therapeutic target [189]. Cetuximab, an EGFR-targeting monoclonal antibody, is approved for treating metastatic CRC [180]. The CRYSTAL trial demonstrated that combining cetuximab with first-line chemotherapy significantly improved progression-free survival (PFS) in patients with KRAS wild-type metastatic CRC [190]. However, resistance remains an unavoidable issue, often arising from mutations in the EGFR, KRAS, and BRAF genes. A mutation at codon 492 of EGFR, which changes serine to arginine (S492R), can alter the conformation of EGFR’s extracellular domain, leading to cetuximab resistance [191]. Additionally, cetuximab is effective only in patients with KRAS wild-type tumors, as KRAS mutations keep the MAPK signaling pathway activated, leading to resistance to EGFR-targeted therapies [192, 193]. BRAF mutations, particularly BRAFV600E, are also implicated in cetuximab resistance, resulting in poor treatment response and prognosis [191].

The Wnt/β-catenin pathway, a critical signaling cascade regulating various biological processes, including embryogenesis, stem cell maintenance, and tissue homeostasis, plays a key role in CRC initiation, progression, and resistance [194, 195]. Forkhead Box M1 (FOXM1) is involved in the malignant behavior of CRC by enhancing Wnt/β-catenin signaling. FOXM1 interacts with Dishevelled 2 (Dvl2), promoting its nuclear translocation and transcriptional activity, thereby driving metastasis and drug resistance [196]. Additionally, LGR5, a marker for cancer stem cells (CSCs) and a Wnt pathway target, contributes to chemoresistance by allowing CSCs to enter a quiescent state and evade drug treatments [197].

Resistance in CRC is closely linked to patient-specific variations and tumor heterogeneity. The mutation burden of resistance-associated genes significantly affects the efficacy of targeted therapies. By investigating the mechanisms behind CRC resistance, new therapeutic targets may be identified. Furthermore, a detailed evaluation of the molecular profiles of individual patients could enable the development of personalized adjuvant treatment strategies, ultimately improving clinical outcomes for advanced CRC patients.

### Prostate cancer

Prostate cancer (PC) is the second most common malignancy among men worldwide, accounting for approximately 15% of all cancer diagnoses in men [198]. In recent decades, the treatment landscape has significantly evolved, with androgen deprivation therapy (ADT) becoming the standard approach for PC patients. ADT works by reducing androgen levels produced by the testes, depriving prostate cancer cells of their primary growth stimulus, leading to tumor regression or slower proliferation [199]. However, the effectiveness of ADT is often limited by the development of resistance. After 18 to 24 months of treatment, many patients progress to castration-resistant prostate cancer (CRPC), which no longer responds to ADT [200].

Resistance to ADT is primarily driven by adaptive changes in the androgen receptor (AR) signaling pathway. Mechanisms include AR amplification, point mutations in the AR gene, alterations in AR co-regulatory molecules, and changes beyond the AR pathway. Overexpression of AR allows tumor cells to survive and grow despite low androgen levels during therapy. In CRPC, increased AR mRNA and protein levels are commonly observed, likely due to epigenetic modifications [201, 202]. AR gene mutations, present in approximately 10–20% of CRPC cases, reduce the receptor’s specificity for testosterone, allowing it to bind other hormones like estrogens and progestins, diminishing the effectiveness of AR antagonists and contributing to chemotherapy resistance [203–205].

Changes in AR co-regulator expression also provide a survival advantage to cancer cells during ADT, promoting progression to CRPC. Co-regulators modulate AR transcriptional activity by acting as coactivators or corepressors [205]. Notably, steroid receptor coactivator-3 (SRC3) plays a critical role in AR activation and CRPC progression [206]. While AR reactivation is central to CRPC progression, alternative pathways also contribute to resistance. Heat shock protein Hsp27, for example, plays a key role in driving resistance. Inhibitors of Hsp27, such as antisense oligonucleotides (OGX-427) and small interfering RNA (siRNA), have shown potential to enhance chemotherapy efficacy. Hsp27 protects specific proteins involved in castration resistance, such as eIF4E, from degradation, promoting tumor survival [207, 208].

Neuroendocrine prostate cancer (NEPC) is a highly aggressive subtype that often develops following prolonged ADT. In NEPC, adenocarcinoma cells transform into neuroendocrine cells, a process driven by complex interactions among genomic, epigenetic, transcriptional, and post-translational changes [209–212]. NEPC is resistant to endocrine therapies, necessitating platinum-based chemotherapy as the first-line treatment. Since its introduction in 2004, docetaxel has become an essential therapy for metastatic castration-resistant prostate cancer (mCRPC) [213]. Clinical studies have shown that combining docetaxel with ADT and radiation significantly improves recurrence-free survival in patients with non-metastatic, locally advanced prostate cancer. However, resistance to docetaxel limits its long-term effectiveness [214]. Chemotherapy resistance in PC arises from various factors, including changes in drug targets, epigenetic modifications, DNA repair mechanisms, inhibition of apoptosis, and the epithelial-to-mesenchymal transition (EMT) process [215].

While endocrine therapy can prolong survival in advanced prostate cancer, the transition to CRPC marks a critical point in disease progression, increasing mortality risk. Resistance to established therapies, including immunotherapy and chemotherapy, remains a significant challenge. A deeper understanding of the dynamic changes in treatment targets and resistance mechanisms is essential for developing innovative treatment strategies. These insights can help identify new therapeutic approaches to improve outcomes for patients with advanced prostate cancer.

### Leukemia

Leukemia is a group of cancers characterized by the uncontrolled growth of hematopoietic stem cells, and it is the most common cancer among children and adolescents [216]. Treatment strategies for leukemia include chemotherapy, radiotherapy, immunotherapy, and hematopoietic stem cell transplantation, with specific approaches tailored to different subtypes [217]. Acute myeloid leukemia (AML), a highly aggressive blood cancer, has a poor prognosis. Standard treatment for AML involves the "3 + 7" regimen, a combination of cytarabine (Ara-C) and anthracycline [218]. Recent advances have shed light on key molecular features that help guide targeted treatments, particularly mutations in the FMS-like tyrosine kinase 3 (FLT3) gene, which is altered in about one-third of AML patients [219, 220].

FLT3 inhibitors have improved treatment outcomes, but resistance remains a major challenge [221, 222]. Resistance can be caused by protective interactions in the bone marrow, mutations in the FLT3 gene, and mutations outside the FLT3 target region. The enzyme CYP3A4, which metabolizes FLT3 inhibitors, is highly concentrated in the bone marrow, reducing the drugs’ effectiveness by lowering their plasma levels [223, 224]. Studies show that using CYP3A4 inhibitors like clarithromycin can help overcome resistance to FLT3 inhibitors like sorafenib [225]. Bone marrow stromal cells also produce FLT3 ligand, which promotes AML cell survival and reduces their sensitivity to FLT3 inhibitors [226].

Mutations in the FLT3 gene, including internal tandem duplications (ITDs) and tyrosine kinase domain (TKD) mutations, further contribute to resistance, especially against type II FLT3 inhibitors [227]. Resistance is also driven by other pathways that compensate for FLT3 inhibition, such as the RAS/MEK/ERK and PI3K/AKT/mTOR pathways [228]. Insights from studies on chronic myeloid leukemia (CML) have helped in the development of next-generation FLT3 inhibitors to improve treatment outcomes in AML patients.

CML is caused by the fusion gene BCR-ABL1, which results from a translocation between chromosomes 9 and 22, forming the Philadelphia chromosome [229, 230]. This gene produces the BCR-ABL1 protein with uncontrolled tyrosine kinase activity, driving CML progression [231]. Tyrosine kinase inhibitors (TKIs), which block this activity, have significantly improved CML outcomes [232]. However, 30% of patients develop resistance, primarily due to BCR-ABL1 mutations or overexpression, which reduces TKI effectiveness [233]. Other resistance mechanisms include abnormal drug transport, activation of alternative pathways, and the persistence of leukemic stem cells [229]. These complexities highlight the need for new therapies to overcome resistance and improve survival.

## Overcoming chemotherapy resistance: therapeutic strategies and future directions

The persistence of chemotherapy resistance continues to challenge the efficacy of cancer treatment, underscoring the urgent need for innovative therapeutic strategies. In this section, we discuss four key approaches to addressing this issue: combination therapies, targeting the tumor microenvironment, advanced drug delivery systems, and personalized medicine. Specifically, combination therapies leverage multiple agents to counteract distinct resistance mechanisms, thereby enhancing therapeutic efficacy. Meanwhile, strategies targeting the tumor microenvironment aim to disrupt its supportive role in fostering resistance. In addition, emerging drug delivery technologies are designed to improve the precision, stability, and bioavailability of chemotherapeutic agents, thereby minimizing systemic toxicity and enhancing efficacy. Finally, personalized medicine focuses on tailoring treatment regimens to the unique molecular and genetic characteristics of individual patients, offering a precision-based approach to overcoming resistance. Collectively, these strategies provide a robust and comprehensive framework for combating chemotherapy resistance, representing critical directions for advancing cancer therapy.

### Combination therapies

Since the first use of chemotherapy to treat cancer, the challenge of limited efficacy with monotherapy has persisted [234]. With the discovery of oncogenic targets in the 1980s, targeted therapies were developed. The emergence of immunotherapy, photodynamic therapy (PDT), and radiotherapy (RT) represents some of the most significant breakthroughs in the field of cancer treatment, have further advanced the field [235–237]. A key focus of current research is how to combine these therapies to overcome the limitations of monotherapy [238]. Cellular signaling pathways are complex, often involving feedback loops and crosstalk between pathways. This can lead to compensatory mechanisms, where inhibiting one pathway activates another. For example, combining BRAF and EGFR inhibitors has proven effective where single-agent therapy fails [239]. Inhibition of BRAF alone triggers a feedback loop that activates EGFR, but using both inhibitors together has shown significant efficacy [240]. The combination of cetuximab (an EGFR inhibitor) and encorafenib (a BRAF inhibitor) has been approved for treating BRAF-mutated colorectal cancer in the U.S. and Europe.

The integration of immunotherapy with other treatments has also shown great promise. Immune checkpoint inhibitors (PD-1, PD-L1, CTLA-4) enhance the immune system’s ability to fight cancer and have been approved for various cancers [235]. Combining checkpoint inhibitors with chemotherapy has demonstrated synergistic effects, improving outcomes. Chemotherapy-induced DNA damage can increase tumor antigenicity by enhancing antigen presentation via NF-κB activation and HLA expression [241]. For example, combining pembrolizumab with chemotherapy has improved survival rates in lung cancer compared to chemotherapy alone [242]. Additionally, combining targeted therapies with immunotherapy enhances the immune system’s ability to recognize and destroy cancer cells. BRAF and MEK inhibitors, for instance, have been shown to increase levels of interferon-γ (IFN-γ) and promote T cell activation, leading to enhanced tumor infiltration by CD8 + T cells [243–245]. The combination of immune checkpoint blockade (ICB) and MAPK inhibitors has been approved for treating melanoma [246].

Combining PDT and RT with chemotherapy has shown promising potential. PDT uses specific light wavelengths to activate intracellular compounds, generating reactive oxygen species (ROS) to destroy tumor cells. It also enhances chemotherapeutic drug permeability and modulates immune cell and cytokine infiltration, impacting distant disease sites. Clinical trials exploring PDT as a chemotherapy adjunct are ongoing [247]. PDT holds promise for enhancing treatment efficacy and overcoming chemotherapy resistance. RT, using high-energy X-rays or gamma rays, is essential for tumor control and improving quality of life [248]. Studies indicate that combining intensity-modulated radiotherapy (IMRT) with adjuvant immune checkpoint inhibitors significantly enhances efficacy, reduces postoperative adverse effects, and improves outcomes [249].

These combination strategies leverage the strengths of each modality, producing synergistic effects that can better address resistance and tumor heterogeneity, often achieving more than either treatment alone.

### Targeting the tumor microenvironment

The tumor microenvironment (TME) is a complex network of cells and extracellular components surrounding tumor cells. It is characterized by a low pH, hypoxia, and metabolic byproducts, and includes endothelial cells, fibroblasts, immune cells, cytokines, growth factors, and the extracellular matrix [250]. The interactions between the TME and tumor cells not only promote tumor growth and invasion but also contribute to drug resistance by reducing drug permeability. Targeting the TME offers new opportunities to overcome resistance. For example, in hepatocellular carcinoma, the macrophage colony-stimulating factor (CSF-1) and its receptor regulate tumor-associated macrophages (TAMs). The CSF receptor inhibitor PLX3397 blocks CSF-1R tyrosine kinase activity and, while it doesn’t reduce TAM infiltration, it shifts TAMs toward the M1 phenotype, inhibiting tumor growth [251].

Improving drug delivery within the TME has also been explored as a way to enhance chemotherapy efficacy. One of the most studied methods is the nanoparticle delivery system, which uses cell-penetrating and tumor-targeting peptides designed to deliver drugs directly to the TME [252]. Understanding the TME’s role in tumor progression and resistance is crucial for developing new therapeutic strategies. Precisely targeting the TME could improve treatment outcomes and offer a promising approach to overcoming resistance in cancer therapy.

### Drug delivery systems

Traditional chemotherapy faces several challenges in cancer treatment, such as reduced therapeutic efficacy due to poor drug targeting and increased toxicity from systemic exposure. To address these issues, drug delivery systems have emerged as a promising solution. Nanotechnology-based drug delivery systems encapsulate chemotherapeutic agents in nanoparticles, optimizing drug distribution and accumulation in the tumor microenvironment while minimizing exposure to healthy tissues [253]. Common nanoparticles include liposomes, micelles, and metal-based nanoparticles. Liposomes, composed of phospholipids, self-assemble into spherical vesicles that can carry therapeutic molecules. Their biocompatibility and safety make them ideal for drug delivery [254]. For example, folate-conjugated liposomes used to deliver 5-fluorouracil (5-FU) in colon cancer have shown increased cancer cell death and reduced tumor volume with lower doses of 5-FU [255]. Pegylated liposomal doxorubicin has also reduced cardiotoxicity compared to free doxorubicin, significantly lowering adverse effects [256]. Some nanoparticle-based systems can overcome resistance mechanisms by inhibiting drug efflux pumps and promoting apoptosis. Cationic nanoparticle complexes encapsulating paclitaxel and the Bcl-2 gene have been shown to inhibit drug-resistant liver cancer cells by disrupting P-glycoprotein (P-gp) drug efflux and activating apoptotic pathways [257]. These systems represent a major advance in overcoming drug resistance, improving both the efficacy and safety of cancer treatments by targeting tumor tissues more precisely and reducing off-target effects.

### Personalized medicine

Personalized medicine (PM) is an innovative medical approach that customizes treatment plans based on an individual’s genetic makeup, environment, and lifestyle [258]. This approach aims to provide more effective care by accounting for individual variability. Advances in next-generation sequencing (NGS) have greatly improved genomic technologies, enabling the identification of actionable mutations in cancer patients. Personalized medicine has the potential to improve survival outcomes, particularly in patients with poor prognoses. A well-known example is the treatment of non-small cell lung cancer (NSCLC) in patients with EGFR mutations. NSCLC patients often develop resistance to first-generation EGFR inhibitors due to secondary mutations like EGFR T790M. The development of third-generation inhibitors, such as osimertinib, specifically targets these mutations [133, 136]. While many tumors share common resistance mechanisms, such as issues with drug transporters or DNA damage repair (DDR), each cancer type has unique molecular targets. Furthermore, individual patients vary significantly, making a one-size-fits-all approach ineffective. Therefore, treatments should be tailored to the molecular characteristics of each patient’s tumor, maximizing therapeutic benefit and minimizing toxicity. As genomic technologies continue to advance, personalized medicine will likely play a key role in overcoming resistance to cancer treatments.

### Future directions and emerging therapies

Chemotherapy resistance arises from a complex interplay of multiple mechanisms, not just a single factor. Future research should focus on understanding and addressing these mechanisms, which include drug efflux, DNA damage repair, apoptosis evasion, epigenetic changes, intracellular drug metabolism, and tumor stem cells. Investigating these pathways will deepen our understanding of chemotherapy resistance and guide the development of more effective treatments. Advances in genomics, proteomics, and metabolomics have greatly facilitated the identification of resistance mechanisms across different cancers. Targeted therapies designed to address specific resistance pathways and immunotherapies that activate the immune system to destroy tumors are offering new strategies to combat resistance.

**Fig. 5:**
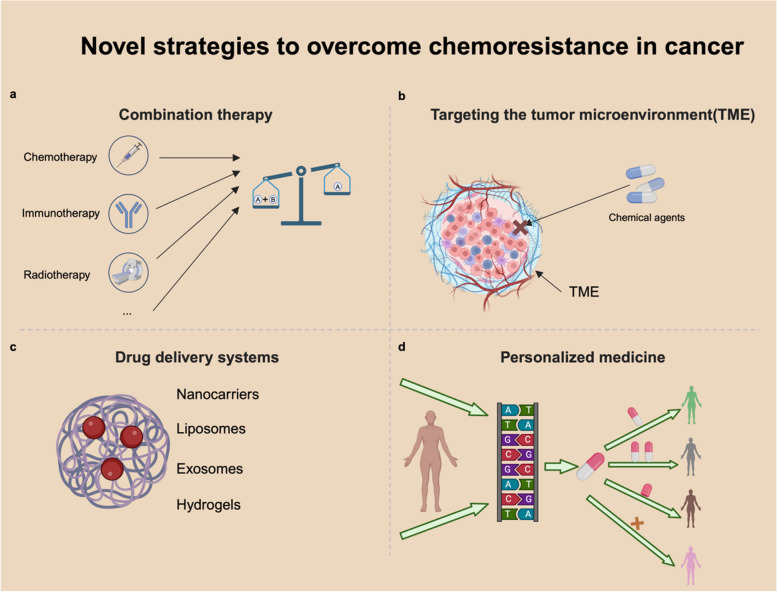
Novel strategies to overcome chemoresistance in cancer. **a** Combination therapy. Combining chemotherapy with other modalities improves therapeutic outcomes. **b** Targeting the tumor microenvironment (TME). The TME serves as a formidable barrier that chemotherapeutic agents must overcome to achieve therapeutic efficacy. **c** Drug delivery systems. Various carriers can enhance drug delivery, addressing issues of poor targeting and increased toxicity. **d** Personalized medicine. Analyzing genetic composition allows for the customization of therapies to achieve the greatest treatment benefit

Combination therapies that integrate targeted treatments, immunotherapies, and traditional chemotherapy are showing great promise, as they exploit the synergistic effects between different modalities. Emerging therapies such as PDT, neural therapies, mRNA vaccines, and biologics are also rapidly progressing, expanding the arsenal against cancer. Going forward, cancer treatment will place greater emphasis on personalized medicine and optimizing drug delivery systems to enhance drug accumulation in tumors while reducing systemic toxicity. With a comprehensive understanding of chemotherapy resistance mechanisms, the field of oncology is poised to deliver more effective and tailored treatments, ultimately improving patient outcomes. (Fig. 5).

## Conclusion

Chemotherapy resistance remains a major challenge in cancer treatment. This review has explored the diverse molecular mechanisms underlying chemotherapy resistance, including DNA damage repair, apoptosis evasion, epigenetic modifications, intracellular drug metabolism, and the role of cancer stem cells. Each of these mechanisms contributes to the complexity of treatment resistance, creating significant obstacles for current therapeutic approaches. Additionally, we have examined resistance mechanisms in several major cancer types, highlighting key molecular targets.

Current efforts to overcome chemotherapy resistance focus on a range of strategies, including targeting multiple resistance pathways. Despite significant advancements, cancer continues to evolve, finding ways to evade the effects of chemotherapy. Future research should prioritize a deeper understanding of the molecular basis of resistance and leverage advanced technologies to identify novel therapeutic targets. Developing combination therapies that address multiple resistance mechanisms simultaneously will be essential. Moreover, improving drug delivery systems and adopting personalized medicine approaches will play a crucial role in reversing chemotherapy resistance. These strategies have the potential to offer patients more effective and tailored treatment options, ultimately improving outcomes in the battle against cancer.

## Data Availability

Not applicable.
